# Hippocampus subfield volumetry after microsurgical or endovascular treatment of intracranial aneurysms—an explorative study

**DOI:** 10.1186/s41747-019-0092-7

**Published:** 2019-03-21

**Authors:** Dennis M. Hedderich, Tim J. Reess, Matthias Thaler, Maria T. Berndt, Sebastian Moench, Manuel Lehm, Tiberiu Andrisan, Christian Maegerlein, Bernhard Meyer, Yu-Mi Ryang, Claus Zimmer, Maria Wostrack, Benjamin Friedrich

**Affiliations:** 1 Department of Diagnostic and Interventional Neuroradiology, Klinikum Rechts der Isar, Technical University of Munich, Munich, Germany; 2 TUM-Neuroimaging Center, Technical University of Munich, Munich, Germany; 3 Department of Neurosurgery, Klinikum Rechts der Isar, Technical University of Munich, Ismaninger Str. 22, 81675 Munich, Germany

**Keywords:** Intracranial aneurysm, Hippocampus, Magnetic resonance imaging, Radiology (interventional)

## Abstract

**Background:**

To study hippocampus subfield volumes in patients after microsurgical clipping (MC) and/or endovascular coiling (EC) of intracranial aneurysms.

**Methods:**

Hippocampus subfield volumetry was performed using FreeSurfer v6.0 in 51 patients (35 females, mean age 54.9 ± 11.9 years, range 24–78 years). Visual inspection of image and segmentation quality was performed prior to statistical analyses. Multiple regression analysis, controlled for age, sex, and side of treatment, was used to assess the impact of prior MC and history of subarachnoid haemorrhage (SAH) on hippocampus subfield volumes (cornu ammonis (CA)-2/3, CA-4, subiculum). Partial correlation analyses were used to assess effect of multiple treatments on hippocampus subfield volumes.

**Results:**

Prior MC was significantly associated with lower hippocampal subfield volumes in MC patients for right and left CA-2/3 (*β* = -22.32 [-40.18, -4.45]; *p* = 0.016 and *β* = -20.03 [-39.38, -0.68]; *p* = 0.043) and right CA-4 (*β* = -17.00 [-33.86, 0.12]; *p* = 0.048). History of SAH was not significantly associated with hippocampal subfield volumes. We observed a higher disease burden in the MC cohort. The number of aneurysms correlated with right-sided hippocampal subfield volumes while the number of treatment interventions did not.

**Conclusion:**

In this explorative study, we found that history of MC was significantly associated with lower volumes in distinct hippocampal subfields, which may be a consequence of a more extensive treatment. This could indicate specific atrophy of CA-2/3 after MC and should motivate hippocampal subfield assessment in larger cohorts.

## Key points

- Hippocampus subfield volumes were measured after intracranial aneurysm repair.
- Prior microsurgical clipping was significantly associated with lower volumes in left cornu ammonis (CA)-2/3 in patients treated with intracranial aneurysms.
- Results could indicate CA-2/3 atrophy after microsurgical aneurysm repair, possibly related to a more extensive treatment.

## Background

Aneurysmal subarachnoid haemorrhage (SAH) is a possibly fatal disease that predominantly affects patients under 60 years of age and may lead to chronic disability [1]. Even if a favourable neurological and functional outcome can be achieved, patients frequently experience impairments of executive functioning, language processing, and cognition as well as psychiatric symptoms such as anxiety and depression [2, 3]. These symptoms potentially affect the patients’ quality of life and are associated with decreased functioning in activities of daily life; thus, a relatively high percentage of patients experiencing these symptoms are unable to return to work [3].

Treatment of intracranial aneurysms is indicated in the acute setting to prevent recurrent SAH and can be achieved either by a microsurgical or an endovascular approach, using either clips or coils to eliminate the aneurysm from the circulation [1]. Due to the increased use of advanced imaging methods of the intracranial arteries, aneurysm detection and thus prophylactic treatment in patients without a history of SAH has become more frequent recently [4]. The decision whether to pursue surgical or endovascular aneurysm occlusion is usually based on individual factors such as the patient’s condition, aneurysm location and morphology.

Both clipping and coiling are widely accepted therapeutic approaches and obviously bear different procedural risks. However, some studies have indicated that patients undergoing surgery are more likely to suffer from impaired cognition and from neuropsychiatric symptoms such as anxiety and depression [5]. A recent study identified postoperative delayed paradoxical depression following surgical repair of unruptured intracranial aneurysms in around 10% of patients, which may be comparable to mild posttraumatic stress disorder and resulted in a reduced rate of patients fully returning to their activities of daily life [6]. There are previous reports hinting towards atrophy of parts of the limbic system, namely the hippocampus in patients after surgical aneurysm repair [7–10].

Taking into account that recent developments in neuroimaging have made it possible to reliably study the hippocampus and its subfields *in vivo* [11, 12], the purpose of this explorative study was to investigate whether the type of treatment or history of SAH could be associated with hippocampal subfield volumes and whether potential changes are related to neuropsychiatric symptoms.

## Methods

### Study population

Patients were recruited between April 2012 and June 2013 from our outpatient clinic as previously described by Wostrack et al. [7]. Inclusion criteria were history of treatment of at least one ruptured or incidental intracranial aneurysm by microsurgical clipping (MC) and/or endovascular coiling (EC), favourable outcome after treatment as measured by a modified Rankin Scale (mRS) score of ≤ 2, and absence of treatment associated impairment and (transient or permanent) peri-procedural complications (*e.g.,* postoperative haemorrhage, infection, thromboembolic events). Exclusion criteria were history of stroke or other neurological disease which may potentially impair quality of life or lead to neurological decline, history of intracranial surgery other than aneurysm occlusion, diagnosis of a current depressive episode or other psychiatric disorders, premorbid mRS ≥ 1, contraindications to magnetic resonance imaging (MRI) such as pacemakers or non-compatible metal implants, age < 18 or ≥ 80 years and absent legal competence.

Patients were stratified in two groups depending on whether an intracranial aneurysm was ever treated by MC or by EC occlusion only. Treatment options for each patient were discussed in interdisciplinary board meetings in order to reach an individual recommendation. Criteria for this decision followed recent guidelines and included aneurysm morphology, presence of perforating vessels, aneurysm location, patient age, and comorbidities [13]. Clinical data was retrieved from the patient files including age, sex, number, and dates of previous treatments, history of SAH (including Hunt and Hess grade if applicable) [14], and total number of intracranial aneurysms.

The initial study was improved by the local ethics committee of the Technical University of Munich (project number 5295/12; March 2012).

### Neuropsychological testing

Patients were evaluated using a standardised neuropsychological test battery, including the Hospital Anxiety and Depression Scale (HADS) and Beck Depression Inventory (BDI)-II which are both widely used in clinical practice and have been applied in studies focusing on post-stroke depression or neurocognitive changes after SAH [3, 15].

### Image acquisition and analysis

All patients underwent structural brain scanning using a 3-T whole-body scanner (Philips Achieva, Philips Healthcare, Best, The Netherlands) using an 8-channel head coil. For further volumetric analysis, a high-resolution three-dimensional (magnetisation-prepared rapid gradient-echo, MP-RAGE) T1-weighted sequence was acquired, with the following technical parameters: field of view FOV 240 × 240 mm, 1-mm isotropic voxel size, repetition time 7.730 ms, echo time 55 ms and flip angle 8°.

Image analysis was performed with the FreeSurfer image analysis suite (version 6.0), which is documented and freely available for download online (http://surfer.nmr.mgh.harvard.edu/) [12, 16, 17]. In this recent FreeSurfer release (v6.0), a newly developed version of the hippocampal segmentation tool has been implemented which is based on a Bayesian model with Markov random field priors [11]. Briefly, the applied parametric segmentation algorithm was developed based on high-resolution (0.13 mm) *ex vivo* MRI scans of the human hippocampus from 15 autopsy samples. These *ex vivo* MRI samples were manually segmented and integrated with *in vivo* T1-weighted images (1-mm resolution) in order to establish an atlas of the hippocampal formation with a new Bayesian inference algorithm to detect local variations in MRI contrast. The algorithm segments 12 different hippocampus subregions, namely hippocampal tail; presubiculum; parasubiculum; hippocampus-amygdala-transition-area; molecular layer; granule cell and molecular layer of dentate gyrus; fimbria; and hippocampal fissure, cornu ammonis (CA)-1, CA-2/3, and CA-4.

Test-retest reproducibility of automated hippocampal subfield segmentation using the FreeSurfer suite was evaluated previously. Marizzoni et al. [18] showed very good volumetric and spatial reproducibility for the subfields CA-2/3, CA-4 and subiculum with a reproducibility error of ~ 2% and a Dice coefficient of > 0.90 [18]. For this reason, the present study was limited to these three main hippocampal subfields per side (CA-2/3, CA-4 and subiculum) for which measurement reliability was previously shown.

Every scan was visually inspected for artefacts and segmentation quality by a neuroradiologist with 5 years of experience (DMH). In case of poor segmentation or if artefacts obscured the hippocampus, the respective hippocampal formations were excluded from further analyses. Subjects with good segmentation quality and absent artefacts of at least one hippocampus formation were included in further analyses. Volumetric data were extracted from FreeSurfer and used for statistical analyses.

### Statistical analyses

Statistical analyses were carried out using SPSS (IBM SPSS Statistics, version 23). Multiple regression analyses were performed for hippocampus subfield volumes (dependent variable) and ‘history of MC treatment’ and ‘history of SAH’, separately. Age, sex, total intracranial volume, and side of treatment served as control variables. Differences between the MC and EC group were tested using Fisher’s exact test (sex, history of SAH), Mann-Whitney *U* test (total number of aneurysms, total number of interventions, Hunt and Hess grade [14] of SAH, total score of BDI-II, HADS) and Student *t* tests (patient age, days between the last treatment and examination). Statistical significance was set at *p* < 0.05, all tests are two-sided; *p* values of post-hoc tests were Bonferroni-corrected for multiple comparisons using the Holm method [19].

## Results

### Study population

Of 63 initial patients, 12 were excluded due to imaging artefacts after visual inspection of hippocampal segmentation accuracy, leading to 51 subjects and 93 hippocampi (48 left hemispheric, 45 right hemispheric) that were included in further analyses. Exclusion of hippocampi due to poor segmentation quality and artefacts did not lead to different distribution of hippocampal sides between the MC and EC groups: 21 and 27 left hemispheric hippocampi and 19 and 26 right hemispheric hippocampi for the MC and EC group, respectively. The age of study participants was 54.9 ± 11.9 years (mean ± standard deviation (SD)) and 35 of 51 subjects were female (68.6%). Median time between the first treatment and MRI was 369 s (range 41–2027), median time between the last treatment and MRI was 299 days (range 1–1057).

History of SAH was positive in 28 (54.9%) out of 51 patients, aneurysms were incidental in the remainder of 23 patients. Hunt and Hess grade was available for 27 patients: grade 1, ten patients (35.7%); grade 2, nine patients (32.1%); grade 3, seven patients (25.0%); and grade 4, one patient (3.6%). Distribution of Hunt and Hess grades was not significantly different between groups (*p* = 0.626). Time between the last treatment and MRI ranged from 1 to 1057 days (median 299 days). First treated aneurysm locations were as follows: anterior communicating artery (*n* = 18, 28.6%), intradural internal carotid artery (*n* = 18, 28.6%), middle cerebral artery (MCA, *n* = 15, 23.8%), and posterior circulation (*n* = 12, 19.0%). Multiple aneurysms (range from 2 to 6) were present in 20 of 51 patients (39.2%). Multiple interventions were performed in 16 patients: two interventions in 12 patients (23.5%), three interventions in 3 patients (5.9%) and four interventions in 1 patent (2.0%). No significant differences regarding scores on BDI or HADS between the EC and MC group were observed. Detailed patient characteristics and distribution between the two groups are given in Table 1.

**Table 1:** Characteristics of study participants according to treatment group

		Microsurgical clipping (*n* = 24)	Endovascular coiling (*n* = 27)	*p*
Males		11 (46%)	5 (19%)	0.068
Age		53.6 ± 10.0	56.0 ± 13.5	0.470
Days between the last treatment and MRI		266 [41–994]	287 [1–1057]	0.406
Number of aneurysms				0.010*
	1	10	21	
	2	7	3	
	3	4	2	
	4	1	1	
	5	1	–	
	6	1	–	
Number of interventions		1.6 ± 0.8	1.2 ± 0.5	0.035*
	1	13	22	
	2	8	4	
	3	2	1	
	4	1	–	
Incidental aneurysms		10 (42%)	13 (48%)	0.780
Presence of subarachnoid haemorrhage		14 (58%)	14 (52%)	0.780
Hunt and Hess grade (*n* = 27)				0.626
	1	5 (35.7%)	5 (35.7%)	
	2	6 (42.8%)	3 (21.4%)	
	3	3 (21.4%)	4 (28.4%)	
	4	0	1 (7.1%)	
	5	0	0	
Beck Depression Inventory total score		12.1 ± 8.1	9.7 ± 9.5	0.186
Hospital Anxiety and Depression Scale	Total	10.7 ± 7.4	8.8 ± 8.0	0.321
	Subscore depression	4.8 ± 3.6	4.0 ± 4.5	0.301
	Subscore anxiety	5.9 ± 4.2	4.7 ± 3.9	0.319

Data are given as mean ± standard deviation, median [range], or number (%); *p* values are given for group differences and marked with an asterisk (*) if statistically significant (*p* < 0.05). *MRI* magnetic resonance imaging

### Multiple regression analyses

**Fig. 1:**
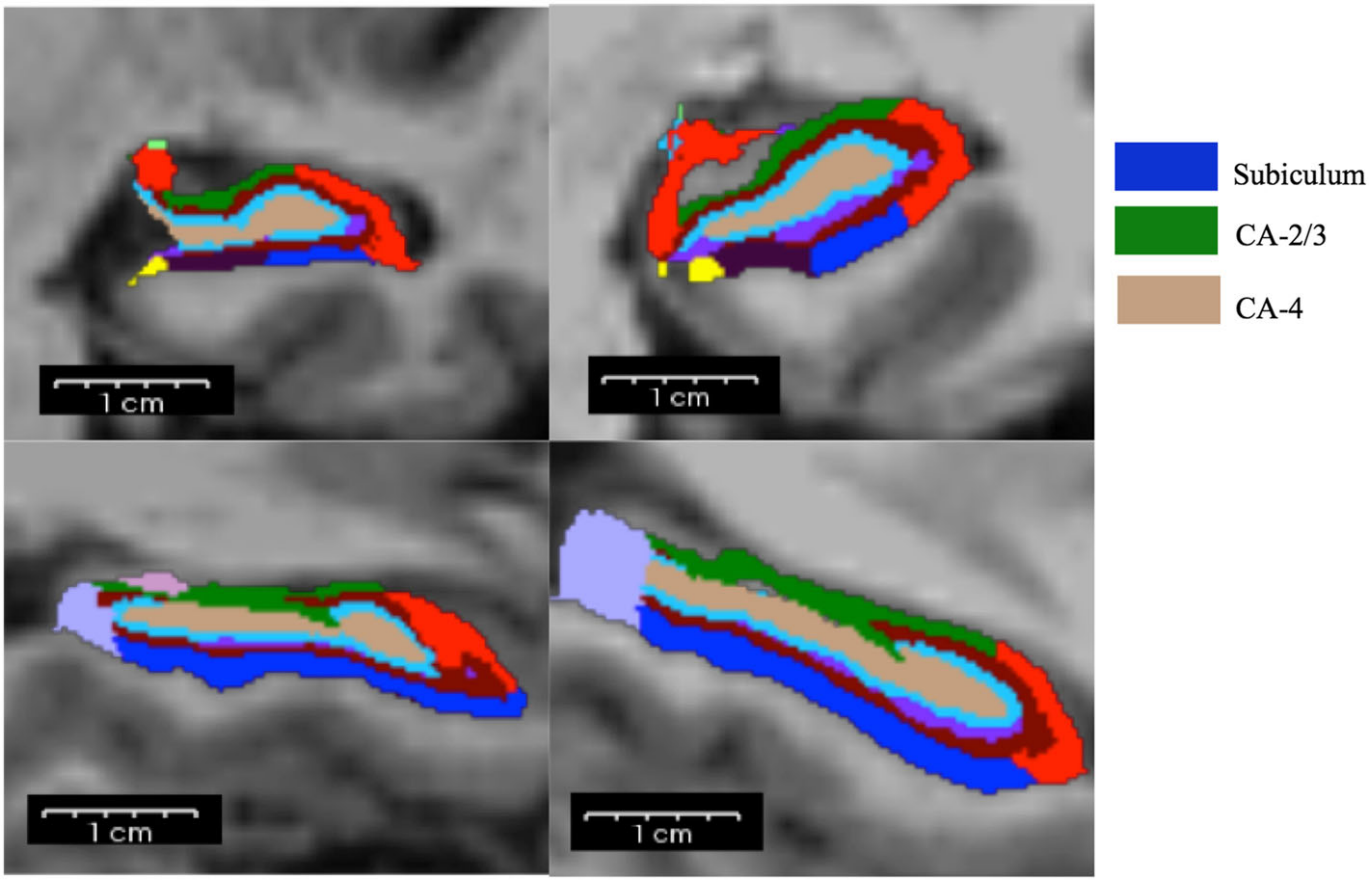
Hippocampal subfield segmentation using FreeSurfer is shown in coronal (upper row) and sagittal planes (lower row). On the left side, the hippocampus of a patient after endovascular aneurysm repair is shown (RAS coordinates: -32.34, 3.80, -17.23). On the right side, the hippocampus of a patient after surgical clipping is depicted (RAS coordinates: -32.45, 2.63, -25.01). *CA* cornu ammonis, *RAS* right anterior superior

Prior MC was significantly associated with lower hippocampal subfield volumes in MC patients for right and left CA-2/3 (*β* = -22.32 [-40.18, -4.45]; *p* = 0.016 and *β* = − 20.03 [-39.38, -0.68]; *p* = 0.043) and right CA-4 (*β* = -17.00 [-33.86, 0.12]; *p* = 0.048). History of SAH was not significantly associated with hippocampal subfield volumes. Detailed results are given in Table 2. An example of hippocampus subfield segmentation is shown in Fig. 1.

**Table 2:** Multiple regression analyses between history of microsurgical clipping and history of subarachnoid haemorrhage (independent variables) in relation with hippocampal subfield volumes (dependent variables)

	Left hippocampus						Right hippocampus					
	CA 2/3		CA 4		Subiculum		CA 2/3		CA 4		Subiculum	
	*β*	*p*	*β*	*p*	*β*	*p*	*β*	*p*	*β*	*p*	*β*	*p*
History of MC	-20.03 (-39.38, -0.68)	0.043	-13.00 (-30.27, 4.27)	0.136	-4.74 (-34.48, 25.00)	0.749	-22.32 (-40.18, -4.45)	0.016*	-17.00 (-33.86, -0.12)	0.048	-5.56 (-36.84, 25.73)	0.721
History of SAH	13.92 (-6.77, 34.60)	0.182	7.51 (-10.77, 25.80)	0.411	-9.07 (-39.89, 21.73)	0.555	13.88 (-5.05, 32.80)	0.146	9.07 (-8.59– 26.73)	0.305	-2.27 (-33.85–29.31)	0.885

Analyses are controlled for age, total intracranial volume, sex, and side of treatment. Unstandardised regression coefficients (*β*) are given with their 95% confidence intervals in parentheses. Statistical significance was set at *p* < 0.05; *p* values that remained significant after correction for multiple comparisons (Holm method) are marked with an asterisk (*). *CA* cornu ammonis, *MC* microsurgical clipping, *SAH* subarachnoid haemorrhage

### Partial correlation analyses

**Fig. 2:**
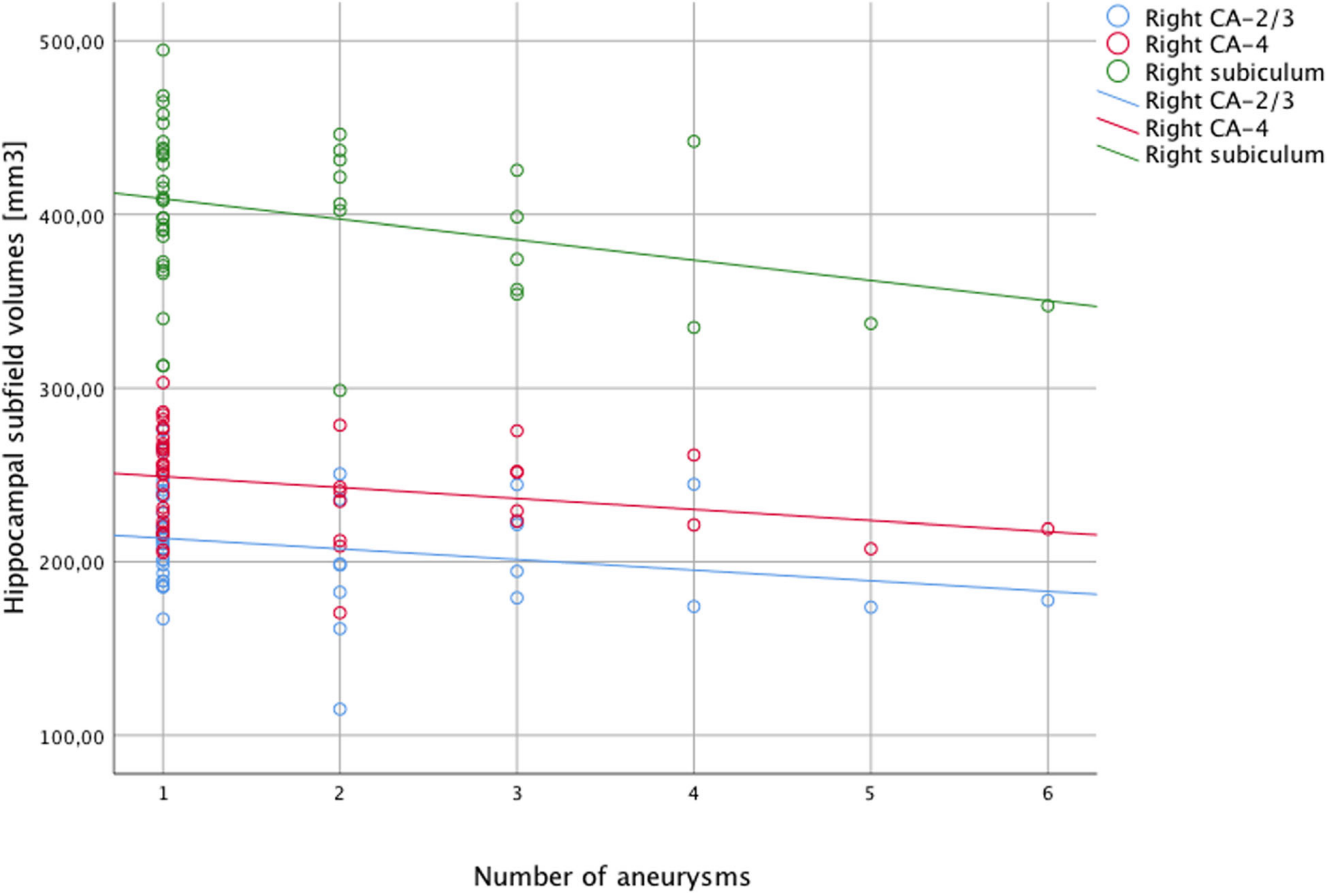
Scatter plot showing volumes of right hemispheric CA-2/3 (blue circles), CA-4 (red circles), and subiculum (green circles) on the *y*-axis and number of total aneurysms (*x*-axis). Linear regression lines are shown in order to illustrate negative correlations between hippocampal subfields and total number of aneurysms. *CA* cornu ammonis

Partial correlation analyses revealed significant negative correlations between the number of intracranial aneurysms and right-sided hippocampal subfields (CA-2/3, *r* = -0.390, *p* = 0.017; CA-4, *r* = -0.415, *p* = 0.011; subiculum, *r* = -0.412, *p* = 0.011) while no significant correlations were found for number of intracranial aneurysms and left-sided hippocampal subfields. Furthermore, no significant correlations were found between the number of treatment interventions and hippocampal subfields on either side. Detailed results are given in Table 3. The correlation between hippocampus subfields and number of intracranial aneurysms is shown in Fig. 2.

**Table 3:** Correlation between the number of treatment interventions and the number of aneurysms

	Left hippocampus						Right hippocampus					
	CA 2/3		CA 4		Subiculum		CA 2/3		CA 4		Subiculum	
	*r*	*p*	*r*	*p*	*r*	*p*	*r*	*p*	*r*	*p*	*r*	*p*
Number of interventions	-0.135	0.389	-0.156	0.319	-0.112	0.474	0.134	0.429	0.031	0.856	-0.067	0.694
Number of aneurysms	-0.255	0.128	-0.290	0.081	-0.246	0.143	-0.390	0.017	-0.415	0.011*	-0.412	0.011*

Partial correlations were controlled for age, sex, total intracranial volume, and side of treatment. Statistical significance was set at *p* < 0.05; *p* values that remained significant after correction for multiple comparisons (Holm method) are marked with an asterisk (*). *CA* cornu ammonis

### Correlation analysis with neuropsychological testing

Partial correlation analysis adjusted for age, gender, side of treatment, and history of SAH revealed no significant correlation between any of the tested hippocampus subfields and BDI, and HADS scores.

## Discussion

In this explorative study, we showed that a history of aneurysm treatment by MC was significantly associated with decreased volumes of distinct hippocampus subfields. However, the MC patient group also had a higher disease burden in terms of number of intracranial aneurysms and underwent more treatment procedures. We also observed a negative correlation between the number of intracranial aneurysms (not number of treatment procedures) and right-sided hippocampal subfields. This may either reflect an effect of more extensive or ‘traumatic’ treatment or be associated with the perceived higher disease burden itself. However, no association between left CA-2/3 volumes and psychometric test results from BDI and HADS scales could be detected.

Endovascular coiling and surgical clipping of intracranial aneurysms have different risks and benefits so that individual treatment suggestions based on multidisciplinary board decisions have to be made considering both the clinical condition and comorbidities of the patient and morphological properties of the aneurysm [4, 20]. Besides immediate focal neurologic deficits associated with intracranial aneurysms and its therapy, both neurocognitive deficits and affective disorders have been described as potential sequelae [2, 3, 6, 21–24]. For middle cerebral artery aneurysms, it was shown that both clipping and coiling are methods with relatively low rates of mortality and morbidity with similar percentages of favourable clinical outcome [20].

Endovascular coiling and surgical clipping both bear similar risk ratios regarding death, bleeding, cerebral ischemia, and functional independence at daily activities for unruptured intracranial aneurysms [4]. However, differences in cognitive outcome measures for patients after clipping compared to endovascular treatment were described [3, 5, 21]. Especially patients with incidental aneurysms bear a relatively high burden of pre-interventional psychiatric symptoms [5]. The hippocampus has been identified to play in vital role in both cognition and affective disorders, which makes this structure especially interesting after intracranial aneurysm repair in light of possible cognitive or affective impairments.

In our study, prior MC was significantly associated with lower volumes in bilateral CA-2/3 and right CA-4, which are key structures of the hippocampus. A part of our study cohort consisted of patients with unruptured intracranial aneurysms that were incidentally detected on MRI scans indicated for different reasons. Among the indications for cranial MRI were conditions such as chronic facial pain, depression, and cognitive decline, which potentially may affect hippocampus volume [5]. However, even if these pre-existing conditions had an impact, the group difference between patients after coiling and clipping could still not be explained.

Basically, there are two major hypotheses regarding a possible impact of treatment type on hippocampus subfield volumes. The first hypothesis is that the open skull surgery itself and the associated trauma to the calvarium, dura mater, and brain parenchyma lead to reactive hippocampal atrophy even if the limbic system itself remains intact. The other hypothesis is that the expectations associated with open aneurysm clipping before and after surgery lead to increased experiences of fear and mood disturbances which might lead to hippocampal atrophy via the neuroendocrine axis and elevated blood cortisol levels.

We have observed a negative correlation for hippocampus subfields and the number of intracranial aneurysms but not for number of interventions. This is interesting because it might suggest that a more extensive or ‘traumatic’ treatment (such as clipping of several intracranial aneurysms during a single procedure) is related to lower hippocampus subfield volumes. Alternatively, one could hypothesise that the psychological stress associated with knowing about a high number of intracranial aneurysms might lead to dysregulation in the limbic system and consecutive hippocampus subfield volume reductions. The association between hippocampal volume and various psychiatric disorders, particularly depression is known from previously conducted research [25]. Especially ‘fear-related’ psychiatric disorders like depression or posttraumatic stress disorder are prone to hippocampal atrophy. The most common theory for this coincidence is that temporomesial atrophy is caused by an increased secretion of glucocorticoids as a neuroendocrine consequence of fear [15]. A recent study on hippocampal shape and cortisol in patients with major depressive disorder found a linear relationship between deformations of the hippocampal shape in CA-1 and CA2/3 subfields with cortisol levels. However, the authors did not find any differences in hippocampal subfield volumes associated with blood cortisol levels [26].

Important limitations to consider for our study are the relatively low number of patients, the retrospective study design, the inclusion of both ruptured, and the unruptured intracranial aneurysm as well as the lack of a control group yielding inherent methodological constraints. The retrospective study design also led to the relatively high number of days between first/last treatment and MRI which can be explained by recruitment of patients during follow-up visits in our outpatient clinic.

These constraints need to be considered, albeit statistical analyses were controlled for possible confounding factors such as age, sex, and total intracranial volume. A major limitation is the heterogeneity of the two treatment groups with MC patients showing both a higher number of treatments and a higher number of intracranial aneurysms. Also, it needs to be considered that by including only patients with good functional outcomes and without transient or permanent treatment-associated impairment, a higher number of EC patients might have been potentially eligible for study enrolment. Additionally, the patients included in this study were not tested for symptoms of posttraumatic stress disorder. The applied neuropsychological batteries focused on depression and more severe symptoms. Thus, mild neuropsychological impairments may have been overlooked. Technically, the imaging and volumetry of the lesioned brain is demanding and parenchymal losses may be the source of segmentation errors. This was addressed by careful visual inspection of the segmented hippocampal subfields.

In conclusion, in this explorative study, we have shown that prior MC treatment of intracranial aneurysms was significantly associated with lower volumes in key subfields of the human hippocampus. However, the treatment groups were heterogeneous in terms of disease burden and we observed a negative correlation between number of aneurysms and right-sided hippocampus subfields. No significant correlations between hippocampus subfields and psychometric test results. In our opinion, these results provide a good rationale for evaluating hippocampus subfields in larger, prospective studies to further elucidate the relationship between disease burden, treatment type, and neuropsychological impairments.

## Abbreviations

BDI: Beck Depression Inventory
CA: Cornu ammonis
EC: Endovascular coiling
HADS: Hospital Anxiety and Depression Scale
MC: Microsurgical clipping
MCA: Middle cerebral artery
MRI: Magnetic resonance imaging
SAH: Subarachnoid haemorrhage
SD: Standard deviation
